# Unidirectional Regulation of Vimentin Intermediate Filaments to Caveolin-1

**DOI:** 10.3390/ijms21207436

**Published:** 2020-10-09

**Authors:** Xuemeng Shi, Changyuan Fan, Yaming Jiu

**Affiliations:** 1 The Joint Program in Infection and Immunity, Guangzhou Women and Children’s Medical Center, Guangzhou Medical University, Guangzhou 510623, China; xmshi@ips.ac.cn; 2 The Center for Microbes, Development and Health, Key Laboratory of Molecular Virology and Immunology, Institute Pasteur of Shanghai, Chinese Academy of Sciences, Shanghai 200031, China; cyfan@ips.ac.cn; 3 Department College of Life Sciences, University of Chinese Academy of Sciences, Yuquan Road No. 19(A), Shijingshan District, Beijing 100049, China

**Keywords:** intermediate filaments (IFs), vimentin, caveolae, caveolin-1 (Cav-1)

## Abstract

Both the mechanosensitive vimentin cytoskeleton and endocytic caveolae contribute to various active processes such as cell migration, morphogenesis, and stress response. However, the crosstalk between these two systems has remained elusive. Here, we find that the subcellular expression between vimentin and caveolin-1 is mutual exclusive, and vimentin filaments physically arrest the cytoplasmic motility of caveolin-1 vesicles. Importantly, vimentin depletion increases the phosphorylation of caveolin-1 on site Tyr14, and restores the compromised cell migration rate and directionality caused by caveolin-1 deprivation. Moreover, upon hypo-osmotic shock, vimentin-knockout recovers the reduced intracellular motility of caveolin-1 vesicles. In contrary, caveolin-1 depletion shows no effect on the expression, phosphorylation (on sites Ser39, Ser56, and Ser83), distribution, solubility, and cellular dynamics of vimentin filaments. Taken together, our data reveals a unidirectional regulation of vimentin to caveolin-1, at least on the cellular level.

## 1. Introduction

All eukaryotic cells contain three major cytoskeletal networks: microfilaments, microtubules, and intermediate filaments (IFs). Vimentin is one of the most well-studied components of IFs, which forms highly dynamic structures in cells [1]. Vimentin has been reported to regulate several endocytic pathways and intracellular vesicle transport. Moreover, vimentin IFs were associated with integrins at focal adhesion sites, and thus involved in mechanical stability, migration, and contraction [2].

Caveolin-1 (Cav-1) is the main structural protein of flask-shaped pinocytic caveolae [3]. Cav-1 is considered to be regulators of multiple essential cellular functions, including endocytic transport, signal transduction, lipid homeostasis, and mechanical transduction [4,5,6].

Increasing studies have confirmed the interaction between caveolae and cytoskeleton, mainly with actin and microtubule networks [7]. For example, circulation of caveolae between the cytoplasmic membrane and the Golgi body is microtubule-dependent [8]. Caveolae can connect mechanical transduction pathways to actin-controlled changes in tension by association with stress fibers [9]. However, the specific interaction and regulation mechanisms between vimentin, major IFs, and Cav-1 remain elusive.

Here, we found that the interaction between vimentin and Cav-1 is unidirectional. Vimentin physically inhibited the motility of cytoplasmic Cav-1 vesicles. In addition, vimentin depletion induced elevated phosphorylation level of Cav-1, and restored the persistent migration and wound healing defects caused by Cav-1 deletion. Whereas, Cav-1 depletion showed no significant effects on the transcriptional, translational, post-translational modification, subcellular expression, and dynamics of vimentin.

## 2. Results

### 2.1. The Subcellular Distribution, Cytoplasmic Motility, and Phosphorylated Level of Cav-1 Are Negatively Regulated by Vimentin Filaments

**Figure 1:**
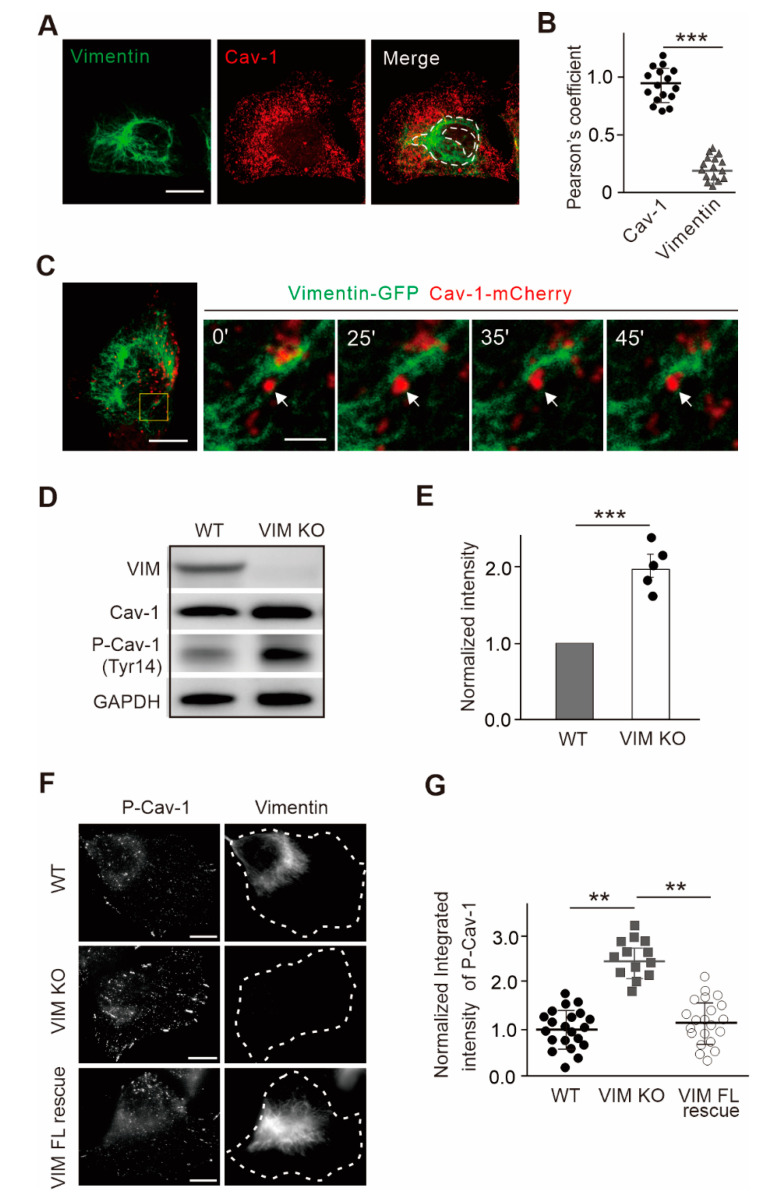
Cav-1 was negatively regulated by vimentin filaments. (**A**) Representative immunofluorescence images showed the subcellular distribution of endogenous vimentin and Cav-1. The white dotted outline showed the perinuclear vimentin-rich region. Bars, 10 µm. (**B**) The correlation analysis indicated by Pearson’s coefficient of the fluorescence signals of endogenous vimentin and Cav-1. The correlation between exogenous expressed Cav-1-mCherry and endogenous antibody stained Cav-1 was used as a positive control. (**C**) Representative images from time-lapse videos (Supplementary Materials) of U2OS cells expressing vimentin-GFP and Cav-1-mCherry. Magnified panels showed representative images at different time points in the yellow box of the left panel, and white arrows indicated the positions of individual Cav-1 vesicle observed in the video (Supplementary Materials). Bars, 10 µm in the left panels and 2 µm in the right magnified panels, respectively. (**D**) The extracts of U2OS cells with wild-type and vimentin-knockout (VIM KO) were subjected to Western blot analysis. (**E**) The quantified relative levels of phosphorylated Cav-1 (P-Cav-1) protein normalized to the internal control GAPDH in wild-type and vimentin-knockout cells from Western blots. The data was from five independent experiments (*n* = 5). (**F**,**G**) The intensity of P-Cav-1 was increased in vimentin-knockout U2OS cells and was able to recover in vimentin full-length (FL) reintroducing cells. In (**F**), it was showed the representative images of wild-type (arrowheads in WT and VIM KO Mix), vimentin-knockout and vimentin-knockout/full-length (arrowheads in VIM KO and FL Mix) cells. In (**G**), it was showed the quantification of normalized relative fluorescence intensities in wild-type (*n* = 21), vimentin-knockout cells (*n* = 13) and full-length rescue cells (*n* = 22). Bars, 10 µm. All the quantified data was presented as mean ± s.e.m. ** *p* ≤ 0.01, *** *p* ≤ 0.001 (*t*-test).

To assess whether caveolae are associated with IFs cytoskeleton, we performed an immunofluorescence experiment to visualize the endogenous vimentin and cellular Cav-1. U2OS cells are much adherent, which are suited for imaging study and show clear endogenous staining of vimentin and Cav-1 [10]. We thus used U2OS cells in this study. It is noted that Cav-1 vesicles were not uniformly distributed throughout the cytoplasm, but were less abundantly expressed in the perinuclear IF-pronounced region. Most Cav-1 vesicles localized either between or at the intersections of the vimentin filaments network (Figure 1A,B), while microtubules or actin filaments showed close connection with Cav-1 vesicles in previous research [11,12,13], which indicated the potential discrepancy regulation of vimentin to caveolae compared to either microtubules or actin filaments.

Regarding our recent work revealing that vimentin IFs function as the barrier for the cellular movements of Cav-1 [10], it is thus worth to further examine the association of vimentin filaments with Cav-1 vesicles by real time. To this end, we applied live cell imaging on cells expressing vimentin-GFP and Cav-1-mCherry. Cav-1 vesicles show very fast dynamics with the average movement velocity about 0.18 µm/s in U2OS cells, while vimentin filaments display much more stable turnover characteristics. Importantly, certain Cav-1 vesicles were observed to run into the vimentin network, deformed the elastic filaments, and then retreated, which occurred within seconds (Figure 1C; Video S1). These results demonstrated that cytoplasmic Cav-1 vesicles are not necessarily co-localized with vimentin cytoskeleton, and the dynamic movement of Cav-1 structures appeared to be restrained by the vimentin IFs network in live cells.

Phosphorylation of Cav-1 on Tyr14 is important for the morphology and migratory behaviors of fibroblasts by Src kinase and Rho GTPases [14,15]. Phosphorylated Cav-1 has also been shown to be involved in activation to the stress response, such as sorting of selected miRNAs into microvesicles to elicit an innate immune response under oxidative stress [16]. We next performed Western blot analysis on wild-type and vimentin-knockout cells. Vimentin depletion resulted in a significant increase in the phosphorylated level of Cav-1, while the total Cav-1 protein was only mildly increased upon vimentin depletion (Figure 1D,E).

Interestingly, vimentin-knockout cells displayed intense focal adhesion and tubular signaling as detected by the staining of phosphorylated Cav-1 on Tyr14 compared to wild-type cells (Figure 1F). We overexpressed cDNA of full-length vimentin in vimentin-knockout cells to construct stable cell lines. The intensity of phosphorylated Cav-1 was increased in vimentin-knockout cells and able to be reversed when introducing full-length (FL) vimentin (Figure 1F,G). The regulation of vimentin to Cav-1 is not restricted to U2OS cells, because immunofluorescence microscopy and Western blot experiments in RNA interference (RNAi)-induced vimentin knockdown human dermal fibroblasts (HDFs) also demonstrated the increased phosphorylated levels of Cav-1. Taken together, these data indicated that intact vimentin IFs regulated the subcellular distribution, motility, and the phosphorylation level of Cav-1.

### 2.2. Depletion of Vimentin Enhances the Motility of Cytoplasmic Cav-1 Vesicles in Hypo-osmotic Culture Condition

**Figure 2:**
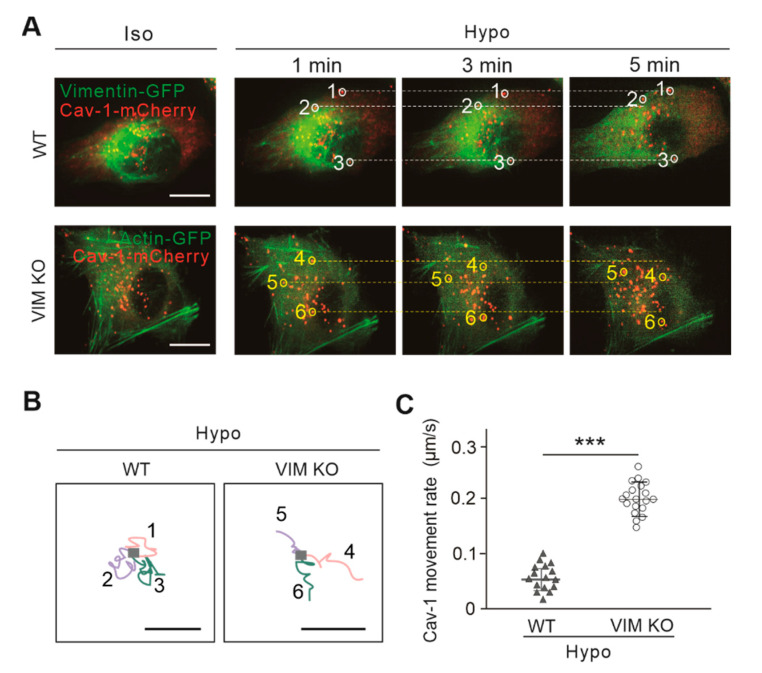
The motility of cytoplasmic Cav-1 vesicles under hypo-osmotic shock was changed with the abolishment of vimentin. (**A**) Time-lapse imaging of U2OS cells expressing Cav-1-mCherry and vimentin-GFP (or expressing Cav-1-mCherry and Actin-GFP in vimentin-knockout U2OS cells) in both normal and hypo-osmotic culture. White and yellow circles indicated discrete Cav-1 vesicles spots in wild-type and vimentin-knockout cells, respectively. Dash lines indicated the positions of Cav-1 vesicles observed in the beginning of the videos (Supplementary Materials). Bars, 10 µm. (**B**) Analysis of trajectories of Cav-1 vesicles in (**A**) upon hypo-osmotic shock. (**C**) Quantification of movement rates of Cav-1-marked vesicles in wild-type cells and vimentin-knockout cells under hypotonic condition. It was showed the Cav-1 movement rate under hypotonic condition in wild-type (*n* = 16) and vimentin-knockout cells (*n* = 19). Bars, 10 µm. The quantified data were presented as mean ± s.e.m. *** *p* ≤ 0.001 (*t*-test).

The movement of Cav-1 vesicles appears to be constrained by the IFs network (vimentin and nestin) in living cells, including U2OS and HeLa cells, and thus results in a mild increased movement rate of Cav-1 vesicles as a consequence of depletion of vimentin [10]. In order to further determine the pathogenic effect of vimentin abolishment on the motility of cellular Cav-1 vesicles, we studied the movement of Cav-1 vesicles under hypo-osmotic stress. Live-cell imaging was performed on cells overexpressing vimentin-GFP and Cav-1-mCherry. It was observed that Cav-1 vesicles in wild-type cells apparently became less motile upon hypo-osmotic stress (Figure 2A). However, after abolishment of vimentin, the movement of Cav-1 vesicles under hypo-osmotic stress was significantly enhanced (Figure 2A). Actin-GFP was co-expressed with Cav-1-mCherry in vimentin-knockout cells to maintain the comparable cell stress caused by transient transfection. Actin-GFP can also be used as a cellular mechanical framework to better characterize the location of Cav-1 vesicles. It is note that, hypo-osmotic stress shows subtle effect to the distribution of actin at least within the initial 5 min [17]. We also only expressed Cav-1-mCherry without vimentin-GFP or actin-GFP to exclude the effects of overexpressing cytoskeletal proteins to the vesicle motility, the results remained the same (data not shown).

According to the previous work, vimentin presented a more spread and diffuse distribution under hypo-osmotic stress [17]. This perhaps explains why, in the presence of vimentin, the motion of Cav-1 vesicles induced by hypo-osmosis is further hindered. Consequently, after depletion of vimentin, the movement of Cav-1 vesicles was moderately enhanced. Under the induction of hypo-osmosis, the trajectory of single Cav-1 vesicle was tracked in vimentin-knockout cells (Figure 2B), which further proved that vimentin-knockout cells presented a freer movement and diffusion trajectory. The motion rate of Cav-1 vesicles was also measured, and showed that hypo-osmosis significantly reduced the motion rate of Cav-1 in wild-type cells, while in vimentin-knockout cells, such a reduction in the motion rate was eliminated (Figure 2C). To sum up, in low osmotic cultures, the depletion of vimentin promotes the movement of Cav-1 vesicles.

### 2.3. Depletion of Cav-1 Does Not Affect the Transcription, Expression, Phosphorylation, Solubility, Cellular Distribution, and Dynamics of Vimentin

**Figure 3:**
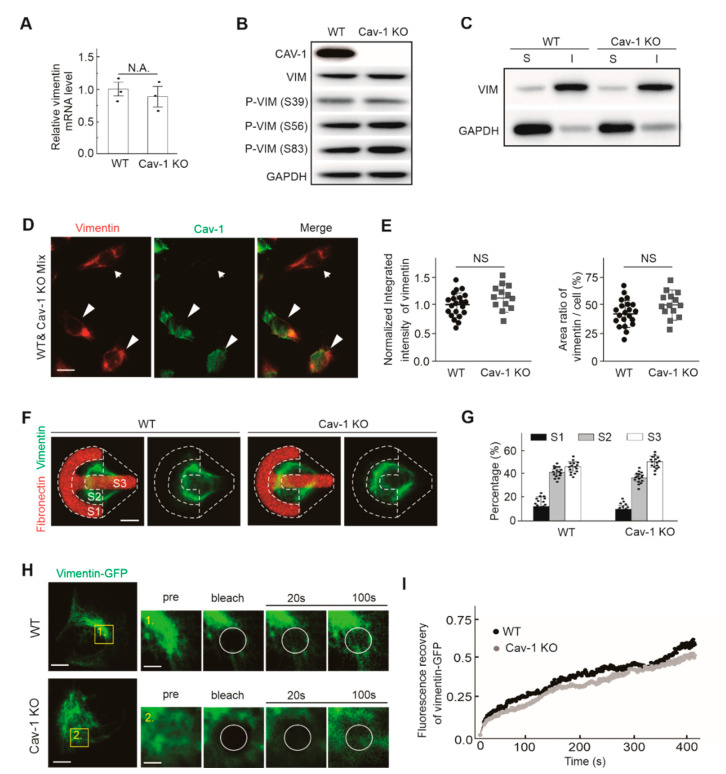
Knockout of Cav-1 had no effect on the expression, phosphorylation, distribution, soluble/insoluble ratio, and dynamics of vimentin in U2OS cells. (**A**) Quantitative RT-PCR (qRT-PCR) analyzed vimentin mRNA levels in wild-type (WT) and Cav-1-knockout (Cav-1 KO) cells. The data was from three independent experiments indicated by black dots. N.A. indicated that the difference between two groups are not significance. (**B**) The extracts of U2OS cells with wild-type and Cav-1-knockout were subjected to Western blot analysis to detect levels of total- and phosphorylated- vimentin. (**C**) Soluble (S) and insoluble (I) extracts of vimentin from wild-type and Cav-1-knockout cells were subjected to Western blot analysis. (**D**) Wild-type and Cav-1-knockout cells were mixed, seeded in the same cover glasses, and stained for immunofluorescence. Representative images showed the subcellular distribution of endogenous vimentin and Cav-1. White arrows indicated Cav-1-knockout cells and white arrowheads indicated wild-type cells. Bars, 50 µm. (**E**) The quantification of vimentin signals in immunofluorescence images. The panel on the left showed normalized relative fluorescent intensities in wild-type and Cav-1-knockout cells. It was shown the quantification of normalized relative fluorescence intensities in wild-type (*n* = 21) and Cav-1-knockout cells (*n* = 13). The panel on the right showed the ratio of vimentin area relative to the cell area. It was shown the ratio of vimentin area in wild-type (*n* = 21) and Cav-1-knockout cells (*n* = 14). (**F**) Localization of the vimentin network in wild-type or Cav-1-knockout cells grown on the fibronectin coated crossbow micropatterns. Cells were divided into three segments S1, S2, and S3 (white dotted outline marked) from the leading edge (left) to the cell rear (right). Bars, 10 µm. (**G**) Percentage distribution of fluorescent signals in segments S1, S2, and S3 in wild-type and Cav-1-knockout cells. It was shown the vimentin distribution in wild-type (*n* = 16) and Cav-1-knockout cells (*n* = 16). (**H**) Representative examples of vimentin-GFP dynamics in wild-type and Cav-1-knockout cells as examined by FRAP. Magnified images showed in the yellow box of the left panels. White circles indicated the photo bleaching regions. Bars, 10 µm in the left panels and 2 µm in the right magnified panels, respectively. (**I**) Averaged recovery curves of the raw FRAP data. The quantified data were presented as mean ± s.e.m. N.S. indicated that the difference between two groups are not significance.

Previously, the influence of Cav-1 expression on vimentin was reported. For example, the expression of vimentin was decreased in Cav-1 silenced PC3 cells [18]. Overexpression of Cav-1 led to down-regulation of E-cadherin and up-regulation of vimentin [19]. Here, we investigated whether knocking out of Cav-1 showed any effects on vimentin. Firstly, Cav-1 CRISPR/Cas9 knockout U2OS cell lines were constructed. The mRNA transcription level of vimentin was quantified by RT-qPCR, and it was found that there was no apparent change in the case of Cav-1-knockout cells (Figure 3A). Regarding the protein level, neither the expression of total vimentin nor the phosphorylated vimentin on site Ser39, Ser56, and Ser83, respectively, was changed in the absence of Cav-1 (Figure 3B). All three serine phosphorylation sites are intimately involved in the reorientation and reorganization of vimentin network [20,21,22].

In addition, the expression of soluble and insoluble vimentin in wild-type cells and Cav-1-knockout cells was analyzed. Vimentin was mainly distributed as insoluble filamentous form in wild-type U2OS cells, and there was little soluble vimentin [23]. The solubility assay showed that there was no significant change in the proportion of soluble and insoluble vimentin (Figure 3C), indicating that Cav-1-knockout did not affect the solubility composition of vimentin. The intracellular expression of vimentin in Cav-1-knockout cells was analyzed by immunofluorescence (Figure 3D). It is noted that there was no obvious difference of vimentin fluorescence intensity between wild-type and Cav-1-knockout cells, and no significant change was observed in the subcellular distribution of vimentin in cells (Figure 3E). Considering that cells plated on coverslips show different morphologies, which makes it difficult to obtain details in subcellular localizations, we plated wild-type and Cav-1-knockout cells on crossbow-shaped fibronectin micropatterns to show regular cell shape and more quantifiable vimentin fluorescence signal [24]. We divided the cells into three segments (from segment 1, the leading edge, to segment 3, the trailing end; see Figure 3F). The experiments did not reveal significant differences for vimentin localization between wild-type and Cav-1-knockout cells (Figure 3G).

In order to further explore whether Cav-1 depletion affects the cellular dynamics of vimentin, we next sought to check the turnover of vimentin with fluorescence recovery after photo bleaching (FRAP), to examine the dynamics of perinuclear vimentin molecules. Vimentin-mEGFP displayed comparable fluorescence recovery in Cav-1 depletion cells compared to wild-type cells (Figure 3H). Quantitative averaged fluorescence recovery curves of vimentin-GFP also supported the similar result (Figure 3I).

Together, elimination of Cav-1 did not influence the transcription, expression, phosphorylation, solubility, cellular distribution, and dynamics of vimentin. Thus, the regulation of vimentin to Cav-1 in U2OS cell appeared to be one way.

### 2.4. Depletion of Cav-1 Does Not Affect the Expression of Vimentin upon Osmotic and Oxidative Stress

**Figure 4:**
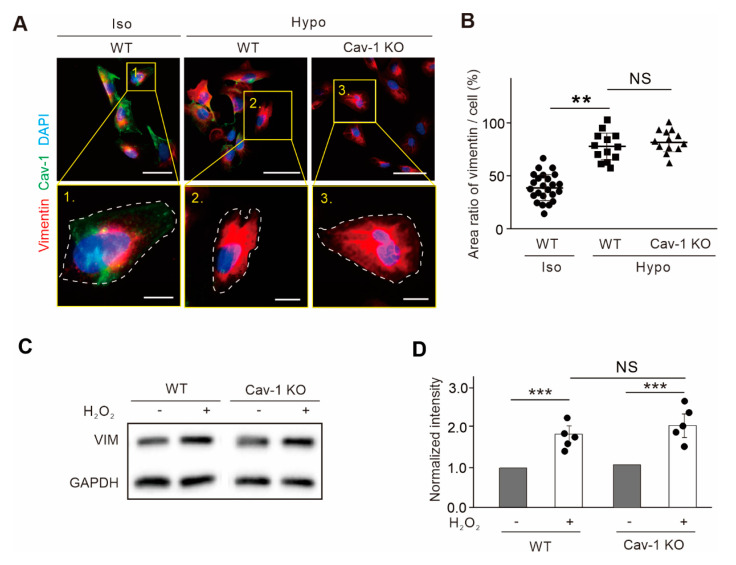
The expression of vimentin under osmotic and oxidative pressure remained the same level with the abolishment of Cav-1. (**A**) Endogenous vimentin IFs in U2OS cells extended under hypotonic stress in both wild-type (WT) and Cav-1-knockout (Cav-1 KO) cells. Magnified images indicated the yellow box in the upper panels. White dash line indicated the outline of the cell. Bars, 50 µm in the upper panels and 10 µm in the lower magnified panels, respectively. (**B**) Quantification of the ratio of vimentin area versus cell area. It was showed the ratio of vimentin area in wild-type (*n* = 21) under isotonic condition, wild-type (*n* = 13) under hypotonic condition, and Cav-1-knockout cells (*n* = 13) under hypotonic condition. (**C**) The extracts from wild-type and Cav-1-knockout cells with or without H_2_O_2_ treatment were subjected to Western blot analysis. (**D**) The quantified relative levels of vimentin protein normalized to the internal control GAPDH. The data was from five independent experiments (*n* = 5). All the quantified data was presented as mean ± s.e.m. ** *p* ≤ 0.01, *** *p* ≤ 0.001 (*t*-test). N.S. indicated that the difference between two groups are not significance.

Cells lacking caveolae showed increased membrane damage when confronted with a variety of physical challenges, such as hypotonic treatment or direct oxidative stress [25]. It is reported that under mild hypotonic conditions, the diameter of caveolae decreased and the invagination of caveolae became flat [26]. These works suggested a regulatory role for caveolae under osmotic stress, and we wondered if this role might correlate with vimentin. The motility of Cav-1 vesicles was improved after knocking out vimentin in the presence of hypo-osmotic shock. Therefore, we wanted to observe whether knockout of Cav-1 would affect the expression of vimentin under osmotic pressure. We observed fluorescence signals of vimentin under isotonic and hypotonic conditions, and selected individual cell for further amplification (Figure 4A). Similarly, immunofluorescence microscopy demonstrated that the endogenous vimentin filaments concentrated to the perinuclear region in U2OS cells in isotonic condition. Upon hypotonic stress, vimentin showed diffuse intracellular distribution with a wider area under low osmotic pressure. Moreover, the distribution of vimentin under hypotonic stress did not significantly change when Cav-1 was knocked out (Figure 4B).

Oxidative stress is another external stimulus, and vimentin is a key sensor of oxidative stress and causes extensive recombination of vimentin network [27,28]. Cav-1 can also be affected by the active substances produced by oxidative stress. For example, Lu et al. found that oxidative stress and apoptosis level in Hashimoto’s thyroiditis cells were higher than that in normal thyroid cells associated with decreased Cav-1 expression [29]. Mougeolle et al. found that when the concentration of non-toxic hydrogen peroxide increased the concentration of active substances in skeletal muscle myoblasts, the level of Cav-1 protein decreased, and the endocytosis was greatly weakened, although caveolae invagination was still able to assemble on the plasma membrane [30]. We wanted to observe whether Cav-1-knockout would affect the expression of vimentin by using H_2_O_2_ to induce oxidative stress. The expression of vimentin was significantly increased regardless of whether Cav-1 is depleted or not (Figure 4C,D). Together, the above two stimulus experiments indicated that Cav-1-knockout did not affect the expression of vimentin neither under osmotic nor oxidative stress conditions.

### 2.5. Deprivation of Vimentin Restores the Defective Cell Migration by Cav-1 Abolishment

Mechanical sensitivity of vimentin and caveolae is associated with cell migration. Vimentin networks connect with integrin at focal adhesion sites, and promote the migration in different cell types [31]. Cav-1 shows a polarized distribution in migrating cells, and changes of Cav-1 expression regulated migration [32,33]. These studies indicate that directional migration and cell polarity require Cav-1 [34]. Thus, we examined the effects of vimentin and Cav-1 on cell migration.

**Figure 5:**
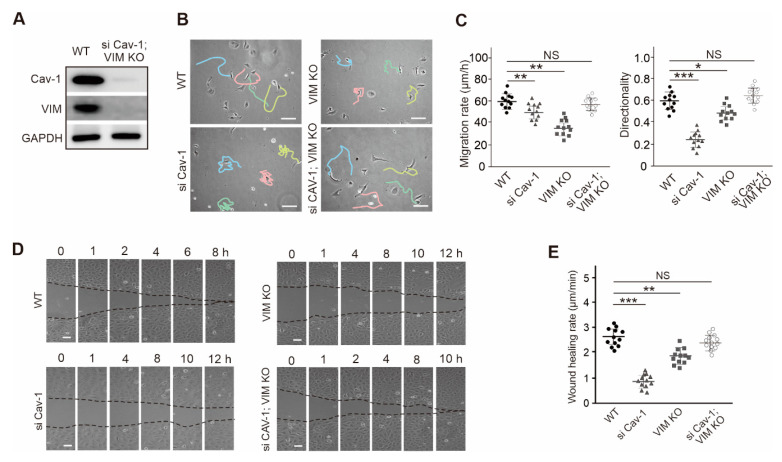
Cell migration ability analysis with the abolishment of Cav-1 or/and vimentin. (**A**) The extracts of wild-type (WT) cells and vimentin-knockout (VIM KO); Cav-1 siRNA (si Cav-1) transfected cells were subjected to Western blot analysis. (**B**) The migration trajectories of WT, si Cav-1, VIM KO, and si Cav-1; VIM KO cells. Solid colored lines indicated the motile tracks of cells. Bars, 50 µm. (**C**) The quantification of the cell migration in (**B**). The panel on the left showed the migration rate and the panel on the right showed the migration directionality. It was shown the migration rate and directionality in wild-type (*n* = 12), Cav-1 siRNA (si Cav-1) transfected cells (*n* = 12), vimentin-knockout (VIM KO) cells (*n* = 12), and si Cav-1; VIM KO cells (*n* = 13). (**D**,**E**) Wound healing assay was performed in WT, si Cav-1, VIM KO, and si Cav-1; VIM KO cells. In (**D**), the representative images in different time point during wound healing were shown. Bars, 100 µm. In (**E**), it was showed the quantitation of the averaged wound healing rate. N.S. indicated that the difference between two groups are not significance. All the quantified data was presented as mean ± s.e.m. * *p* ≤ 0.05, ** *p* ≤ 0.01, *** *p* ≤ 0.001 (*t*-test).

Considering the potential compensatory effects of double gene knockout during cell passaging, which might lead to artificial changes, we used siRNA to transiently knockdown Cav-1 in vimentin-knockout cells, and Western blots verified that siRNA did decrease the protein expression level of Cav-1 (Figure 5A).

We used cell lines with the depletion of vimentin or/and Cav-1, although we did not find that the deletion of Cav-1 affected the expression of vimentin, or intracellular localization, but the deprivation of vimentin or Cav-1, cell migration ability decreased (Figure 5B). It is interesting to note that after abolishment of the two proteins simultaneously by silencing Cav-1 in vimentin-knockout cells, the ability of cell migration recovered, including both the migration rate and directionality (Figure 5C). It is worth mentioning that the vimentin, Cav-1 double knockout cell lines we generated also displayed similar restored migration (data not shown).

At the same time, we also proved this point in the wound healing experiment. Deletion of Cav-1 or vimentin significantly reduced the healing rate. But after silencing Cav-1 in vimentin-knockout cells, the wound healing rate was obviously restored (Figure 5D,E). Together, our results suggested that synchronously down-regulation of Cav-1 and vimentin display more dynamic motility capacity than abolishing one of them with yet an unexplored mechanism.

## 3. Discussion

**Figure 6:**
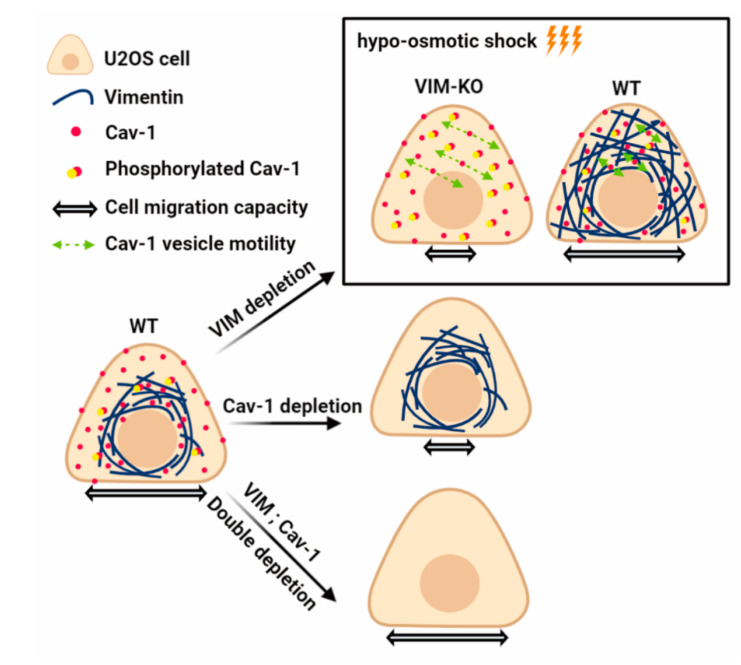
Working model of the relationship between vimentin and Cav-1.

In this study, our main findings include: (**1**) Vimentin and Cav-1 show mutually exclusive subcellular expressions, and vimentin filaments inhibit the cytoplasmic motility of Cav-1 vesicles. (**2**) Absence of vimentin induces dramatical phosphorylation of Cav-1 on site Tyr14 and (**3**) restores the mobility and orientation of damaged cells caused by the absence of Cav-1. (**4**) In addition, removal of vimentin further restores the intracellular activity of Cav-1, which is reduced upon low osmotic shock. (**5**) Conversely, Cav-1 deletion has no effect on the expression, phosphorylation, distribution, solubility, or cellular dynamics of vimentin filament. Taken together, our data show that vimentin affects Cav-1, but not the other way around (Figure 6).

We revealed that the Cav-1 phosphorylation level was significantly improved by vimentin-knockout. Cav-1 phosphorylates at a conserved tyrosine Tyr14 under a variety of stimuli, including mechanical stress, growth factors, oxidative stress, and bacterial invasion [35]. Internalized phosphorylated Cav-1 can form a signal platform on the early endosomes and recruit downstream signal components [36]. Phosphorylation of Cav-1 is associated with transcriptional upregulation of key caveolae components through the EGR1 pathway [37]. Other potential pathways screened by phosphorylated CAV-1 interaction include TRAF2 (tumor necrosis factor (TNF) receptor-associated factor 2) and CSK (C-terminal Src kinase) [38,39] and RhoA-GEF [40]. It is worth noting that in our previous study, the absence of vimentin induced the phosphorylation of microtubule-associated GEF-H1 on site Ser886, thereby promoting RhoA activity and actin stress fiber assembly [41]. Here, the phosphorylated Cav-1 was up-regulated combined with the knockout of vimentin, in line with above studies, it proposed that the phosphorylated Cav-1 participated in RhoA-GEF signaling possibly through vimentin. Our previous study on U2OS cells showed that there was a combination of Cav-1 and vimentin, and vimentin could resist the cytoplasmic movement of Cav-1, acting as a “cage” [10]. It is likely that vimentin, as part of the cytoskeleton, regulates the phosphorylation of Cav-1 and the movement of caveolae by interacting with other cellular components. However, as the main protein component of the caveolae, knockout of Cav-1 did not affect vimentin. This one-way regulation indicates a possible speculation that vimentin acts as superior, whereas Cav-1 acts as subordinate.

Both vimentin and caveolae have been reported to be involved in buffering processes when cells are under stress conditions (for example, osmotic shock and oxidative stress). It thus makes sense to explore whether and how they interact under stress. Hypo-osmotic stress leads to increased IP_3_ levels through the hydrolysis of phosphatidylinositol 4, 5-diphosphate (PIP_2_), which leads to the release of calcium from ER via the IP_3_ receptor (IP_3_R), then increases cytoplasmic calcium-activated vimentin cutting through calpain [42,43]. Caveolae is also involved in this process, and caveolae directly affects Ca^2+^ signals mediated through the Gαq/PLCβ pathway by binding of Cav-1 to Gαq [26]. Furthermore, both Cav-1 and oxidative stress are closely related to tumorgenesis and development. Different promoters and inhibitors of oxidative stress can regulate the occurrence and progression of tumors by regulating the expression level of Cav-1 [44,45]. Rearrangement of vimentin under oxidative stress may also be associated with tumor formation [46]. We observed here that the rearrangement of vimentin under hypotonic stress hindered the movement of Cav-1 vesicles, but we did not find a significant correlation of protein expression between vimentin and Cav-1 under oxidative stress.

With the simultaneous action of vimentin-knockout and Cav-1 siRNA, the inhibition of cell persistent migration caused by individual deletions of vimentin and Cav-1 is restored. After analyzing the reasons, although the mobility and orientation of cell motility returned to the original level with the simultaneous deletion of vimentin and Cav-1, it was possible that the simultaneous deletion of vimentin and Cav-1 caused compensatory enhancement of other potential pathways affecting cell motility, leading to the recovery of migration phenotypes, and is worthy for future study.

In conclusion, here, we discovered one-directional regulation of Cav-1 by vimentin, revealed the negative regulation of vimentin filament to Cav-1, and laid a foundation for further understanding of their functions in cell migration and stress response.

## 4. Materials and Methods

### 4.1. Cell Culture and Transfections

Human osteosarcoma (U2OS) cells (kind gift from Pekka Lappalainen (Helsinki, Finland)) were maintained in high glucose (4.5 g/L) Dulbecco’s modified Eagle’s medium (DMEM) (BE12-614F, Lonza, Basel, Switzerland) supplemented with 10% fetal bovine serum (10500-064, Gibco, Waltham, MA, USA), 10 U/mL penicillin, 10 mg/mL streptomycin, and 20 mM L-glutamine (from 100× concentrate, Gibco, Waltham, MA, USA, later referred as complete DMEM) at 37 °C in humidified atmosphere with 5% CO_2_. Vimentin-knockout U2OS cells were generated using CRISPR/Cas9 methods [24]. Vimentin-knockout and Cav-1-knockout U2OS cells were generated based on pSpCas9 (BB)-2A-GFP vector (a gift from Feng Zhang, #48138, Addgene, Cambridge, MA, USA). Guide sequences were selected based on CRISPR Design Tool (crispr.mit.edu). Then, 5′-CAACGACAAAGCCCGCGTCG-3′ for vimentin-knockout and 5′-AGTGTACGACGCGCACACCA-3′ for Cav-1-knockout. Transfected cells were detached 24 h post-transfection, suspended into complete DMEM with 20 mM HEPES and sorted with FACS Aria II (BD Biosciences, San Jose, CA, USA), using on low intensity GFP-expression pass gating, as single cells onto 96-well plate supplemented DMEM containing 20% FBS and 10 mM HEPES. CRISPR clones were cultivated for two weeks prior selecting clones with no discernible vimentin or Cav-1 protein expression. Transient transfections were performed with Fugene HD (Promega, Madison, WI, USA) according to manufacturer’s instructions using 3.5:1 Fugene to DNA ratio and 24 h incubation prior fixation with 4% paraformaldehyde (PFA) in phosphate buffered saline (PBS).

### 4.2. Plasmids and siRNA Oligonucleotides

Cloning strategy of constructs expressing vimentin full length without tag are described in [47,48]. Vimentin-GFP was kind gift from John Eriksson (Turku, Finland). Actin-RFP was kind gift from Pekka Lappalainen (Helsinki, Finland). Cav-1-mCherry (#27705) was bought from (Addgene, Watertown, MA 02472, USA). All the plasmids acquired were sequenced and compared with the endogenous staining by respective antibodies for verification. AllStars Neg. Control siRNA (Qiagen, Hilden, Germany)) was used as a control siRNA. Primers for siCav-1 were 5′-CCCUAAACACCUCAACGAU-3′ and 5′-AUCGUUGAGGUGUUUAGGG-3′. After 72 h, cells were lysed to determine protein knock down by Western blot analyses or experiments were started.

### 4.3. Immunofluorescence Microscopy

Briefly, U2OS cells were fixed with 4% PFA-PBS for 20 min at room temperature (RT), washed three times with 0.2% BSA in Dulbecco’s phosphate buffered saline, and permeabilized with 0.1% Triton X-100 in PBS for 4 min. Both primary and secondary antibodies were applied onto cells and incubated in RT for 1 h. All data were obtained with Leica TCS SP8 confocal microscopes with a 63x oil or glycerol objectives. The following primary antibodies were used: vimentin mouse (dilution 1:100; Sigma-Aldrich Corp., St. Louis, MO, USA, #V6389); Cav-1 rabbit D46G3 (dilution 1:100; Cell signaling, Beverly, MA, 01915, USA, #3267); Phospho-Cav-1 (Tyr14) rabbit (dilution 1:200; Cell signaling, Beverly, MA, 01915, USA, #3251). Secondary antibodies were conjugated to Alexa Fluor 488 or 568 (Invitrogen, Carlsbad, CA, USA).

### 4.4. Live-cell Imaging

After transient transfection, the cells were incubated for 24 h and re-plated prior to imaging on 10 μg/mL fibronectin (Roche, Basel, Switzerland)-coated glass-bottomed dishes (MatTek Corporation, Ashland, MA, USA)π. The time lapse images were acquired with 3I Marianas imaging system (3I intelligent Imaging Innovations, Denver, CO, USA), consisting of an inverted spinning disk confocal microscope Zeiss Axio Observer Z1 (Zeiss, Oberkochen, Germany) and a Yokogawa CSU-X1 M1 confocal scanner. Then, 63x/1.2 WC-Apochromat Corr WD = 0.28 M27 objective (Zeiss, Oberkochen, Germany), appropriate filters, heated sample environment (37 °C), and CO_2_ control were used. SlideBook 8.0 software (3I intelligent Imaging Innovations, Denver, CO, USA) was used for the image acquirement with the acquire frequency as 1 s per frame. Neo sCMOS (Andor, Belfast, Northern Ireland) camera was used for image recording. Fluorescence intensity along time was averaged by Imaris 9.0 to compensate bleaching.

### 4.5. Fluorescence Recovery in Photobleaching (FRAP)

Wild-type U2OS cells were transfected with vimentin-GFP, and incubated for 24 h. Images were acquired with 3I Marianas imaging system (3I Intelligent Imaging Innovations) as mentioned in live-cell imaging. Five pre-bleach images were acquired followed by bleaching scans with 100% intensity laser lines over the region of interest. Recovery of fluorescence was monitored 50 times every 200 ms and 300 times every 1 s. The intensity of the bleached area was normalized to a neighboring non-bleached area. Mean scatter plots were calculated from different FRAP experiments and the means and standard deviations were calculated.

### 4.6. Western Blotting

Cells were washed with cold PBS, scraped, and lysed in RIPA (Laemmli Sample Buffer). Protein concentrations were measured using Bradford reagent (Sigma-Aldrich). Then, 5% milk was used for blotting. HRP-linked secondary antibodies were used and chemiluminescence was measured after applying ECL Western blotting reagent (AmershamTM, GE Healthcare, Buckinghamshire, HP6 5AY, UK). The same vimentin and Cav-1 antibodies in immunofluorescence were used in Western blot. In addition, vimentin (p-ser39) rabbit monoclonal (dilution 1:1000; Cell signaling, #13614), vimentin (P-ser56) rabbit polyclonal (dilution 1:1000; Cell signaling, Beverly, MA, 01915, USA, #3877), vimentin (P-ser83) rabbit polyclonal (dilution 1:1000; Cell signaling, Beverly, MA, 01915, USA, #3878), and GAPDH mouse polyclonal (dilution 1:1000; Sigma-Aldrich Corp., St. Louis, MO, USA, #G8795) were used.

### 4.7. Solubility Assay

Cells were washed with ice-cold PBS and then collected by centrifugation at 8000× g for 5 min. The resulting pellets were incubated at 37 °C for 30 min in buffer containing 1% Nonidet P-40, 10% (*v*/*v*) glycerol, 20 mM N’-a-hydroxythylpiperazine-N’-ethanesulfanic acid (HEPES) (pH 7.4), 150 mM NaCl, 2 mM sodium orthovanadate, 2 mM molybdate, 2 mM sodium pyrophosphate, and protease inhibitors. The soluble (disassembled) and insoluble (assembled) fractions were collected after centrifugation at 2100× g for 30 min at 4 °C and assessed by immunoblot analysis using anti-vimentin antibody.

### 4.8. Wound Healing Assay

Cells were seeded in a 6-well cell culture plate with a cell density of 25,000/cm^2^ and cultivated at 37 °C in 5% CO_2_ overnight. Subsequently, the cell monolayers were scratched with a sterile 0.2 mL pipette tip to create linear wounds and washed with PBS to remove detached cells. Cells were incubated in a serum-free medium to eliminate cell proliferation and then observed by optical microscopy. The migration rates were measured using ImageJ.

### 4.9. Hypo-Osmotic Shock

The hypo-osmotic medium was made by using complete growth medium diluted appropriately in deionized water (dilution 1:9 to obtain 30 mOsm).

### 4.10. Oxidative Stress

Prepare H_2_O_2_ stock solution in deionized water. Store the solution away from light and keep it at 20 °C until use. All working solutions were freshly prepared in the medium before treatment. Before treatment, it was incubated in a CO_2_ incubator for 24 h, and then treated with 0.1 mM H_2_O_2_.

### 4.11. Statistical Analysis

Statistics were performed with Excel (Microsoft, Redmond, WA, USA) and SigmaPlot 13.0 (Systat Software Inc., Chicago, IL, USA). For data following normal distribution, Student’s two-sample unpaired *t*-test was used. If data did not follow normal distribution, Mann–Whitney *u*-test for two independent samples was conducted.

## Supplementary Materials

The following are available online at https://www.mdpi.com/1422-0067/21/20/7436/s1.

## Abbreviations

Cav-1	Caveolin-1
IFs	Intermediate filaments
VIM	Vimentin
ER	Endoplasmic reticulum
IP_3_	Inositol triphosphate 3
PLCβ	Phospholipase Cβ
